# A novel DNA and protein combination COVID-19 vaccine formulation provides full protection against SARS-CoV-2 in rhesus macaques

**DOI:** 10.1080/22221751.2021.1887767

**Published:** 2021-03-01

**Authors:** Yuzhong Li, Yanwei Bi, Hongjian Xiao, Yueting Yao, Xiaojuan Liu, Zhengrong Hu, Jinmei Duan, Yaoyun Yang, Zhihua Li, Yadong Li, Heng Zhang, Chen Ding, Jianbo Yang, Haiwei Li, Zhanlong He, Longding Liu, Guangnan Hu, Shuying Liu, Yanchun Che, Shixia Wang, Qihan Li, Shan Lu, Wei Cun

**Affiliations:** a Institute of Medical Biology, Chinese Academy of Medical Sciences & Peking Union Medical College, Kunming, People’s Republic of China; b Department of Medicine, University of Massachusetts Medical School, Worcester, MA, USA; c SYL-Consulting, Thousand Oaks, CA, USA

**Keywords:** SARS-CoV-2, spike glycoprotein, DNA vaccine, protein vaccine, non-human primate

## Abstract

The current study aims to develop a safe and highly immunogenic COVID-19 vaccine. The novel combination of a DNA vaccine encoding the full-length Spike (S) protein of SARS-CoV-2 and a recombinant S1 protein vaccine induced high level neutralizing antibody and T cell immune responses in both small and large animal models. More significantly, the co-delivery of DNA and protein components at the same time elicited full protection against intratracheal challenge of SARS-CoV-2 viruses in immunized rhesus macaques. As both DNA and protein vaccines have been proven safe in previous human studies, and DNA vaccines are capable of eliciting germinal center B cell development, which is critical for high-affinity memory B cell responses, the DNA and protein co-delivery vaccine approach has great potential to serve as a safe and effective approach to develop COVID-19 vaccines that provide long-term protection.

## Introduction

The COVID-19 pandemic has caused over 100 million cases of the novel coronavirus and over 22 million deaths globally. While public health measures such as social distancing have played important roles in controlling local outbreaks, the continued spread of COVID-19, especially in remote and underdeveloped areas around the world, only extends the further threat of the pandemic. In addition, many countries have experienced new waves of transmission even after original outbreaks are brought under control. More definitive large scale public health measures like vaccines are the only hope for controlling the global COVID-19 pandemic [1, 2].

Over a dozen COVID-19 vaccines have entered Phase III clinical studies to establish efficacy for large scale public use. Several leading candidates are using novel vaccine platforms such as viral vector [3–7] or mRNA [8–12] approaches, which showed exciting levels of protection efficacy in reports from completed Phase III studies [13, 14]. They have received or are expected to receive Emergency Use Authorization (EUA) by respective regulatory agencies. While their short-term safety has been established, the safety profiles of these vaccines in the long-term, as well as in larger and globally- diverse populations, have yet to be established. One other major type of COVID-19 vaccines under development is the inactivated vaccine approach [15–19]. Although no similar findings have been reported from the current inactivated COVID-19 vaccines, possible adverse events have been observed in the past with this type of vaccine [20, 21]. There are also potential biosafety issues associated with the need to produce large stocks of live SARS-CoV-2 viruses before inactivation. Inactivated vaccines are usually unable to induce cytotoxic T cell immunity (CTL). Traditional inactivated vaccines do not include adjuvants, but some COVID-19 vaccines have added adjuvant to further improve the immunogenicity [22]. At the same time, reports suggest that the SARS-CoV-2 infections may not lead to long-lasting immune responses and that some recovered patients may be re-infected again by the same virus [23–25]. Therefore, it is highly desirable to develop COVID-19 vaccines that are highly immunogenic and elicit long-lasting immunity. It is currently unknown whether any of the leading COVID-19 vaccines can meet such an objective. Additional novel approaches are needed to further enrich the COVID-19 vaccine pipeline to both provide a second generation of practical vaccines and learn more about the unique contributions of different technology platforms.

In this study, we develop a unique subunit COVID-19 vaccine concept by combining the S full-length DNA plasmid and S1 recombinant protein to deliver them at the same time. This concept design is based on a significant body of literature accumulated over the past two decades, including our own work, that demonstrates the effectiveness of the DNA vaccine modality. In vivo production of encoded antigens from DNA immunization activates the endogenous antigen processing and presentation pathway to effectively trigger helper T cell responses, which is critical for B cell development [26]. DNA-primed specific B cells can be further expanded with the addition of a protein component to produce a large amount of desired antibodies. In this study, our novel Covid-19 vaccine design is demonstrated to be more effective in the elicitation of higher immune responses, including neutralizing antibodies and T cell responses, than the use of either DNA or protein component alone. This combination vaccine was also able to elicit full protection against the challenge of SARS-CoV-2 in a non-human primate (NHP) model, which has not been achieved in previous reported COVID-19 vaccine studies in similar NHP models [6, 27–30].

## Materials and methods

### DNA vaccine construction and production

The wildtype and codon optimized SARS-CoV-2 spike full length gene sequences (S-FL-wt and S-FL-opt) were commercially synthesized based on the Wuhan-Hu-1 (GenBank: MN908947). The soluble S ectodomain gene sequence (S-dTM-opt) was generated from the S-FL-opt sequence using the oligomers w1404-TACCGAGCTCGGATCCGCCACCAT and w1406-GATATCTGCAGAATTCTCAAGGCCACTTGATGTACTGCTCG. All three inserts (S-FL-wt, S-FL-opt and S-dTM-opt) were individually subcloned into the mammalian expression plasmid pcDNA3.1+ between BamHI and EcoRI by In-Fusion cloning technology (TAKARA Bio). These S-expressing DNA vaccine plasmids were purified from E. coli DH5α using the endotoxin-free plasmid Maxi kit (Qiagen, USA). All plasmid sequences were confirmed by Sanger DNA sequencing.

The DNA vaccine pCW1093 was produced by subcloning the above S-FL-opt insert into the DNA vaccine vector pSW3891 which, as previously reported, can be used in humans [31]. The insert was amplified from the S-FL-opt template by using the oligomers w1477-TCCATGGGTCTTTTCTGCAGTCACCGTCCAAGCTTGCAATCGCCACCATGTTCGTGTTCCT and w1479-GGGATTGCGAGGATCCTTATCATGTGTAGTGGAGCTTCACG and fused into linearized pSW3891 at PstI and BamHI sites. The pCW1093 plasmid was transformed into competent E. coli and single clones were picked up and amplified to produce the final master seed lot (MSL) and working seed lot (WSL). The pCW1093 DNA plasmid used in the non-human primate challenge study was produced under conditions required by the current good manufacturing practices (cGMP) regulation. Bacteria from WSL were gradually expanded to the fermenter and the pCW1093 DNA plasmids were released from final fermentation bacteria pellet by alkaline lysis. The supercoiled plasmid DNA was further purified by filtration, chromatography and ultrafiltration. The plasmid DNA final products (FP) were tested and buffered by saline solution (pH 7.2) for immunization use.

### S1 protein production and use

Codon optimized gene sequence encoding for S1 protein was subcloned into the mammalian expression vector for in vitro production of recombinant S1 protein for research study applications, and a His-tag was added to the C-terminal of S1 protein for the purpose of purification. The Expi293 cells (Invitrogen, US) were transfected with the S1-expressing plasmid, the supernatant of cell culture was harvested on Day 5, and the S1 protein was purified by HisTrap HP column. The quality was verified by SDS-PAGE and Western blot analysis before being used for immunization and ELISA study purposes. For immunization, S1 protein was absorbed with adjuvant aluminum hydroxide (Brenntag Biosector, Frederikssund, Denmark) at a ratio of 1:10 (w/w).

### Western blot analysis

S-expressing DNA vaccines were tested for their in vitro expression in transiently transfected 293 T cells using polyethylenimine (PEI) as the transfecting agent as previously reported [32]. At 72 h after the transfection, culture supernatants or cell lysates were subject to Western blot analysis with a rabbit polyclonal serum L295-IV specific for the S protein of SARS-CoV-2 virus as the detecting antibody. Similarly, recombinant S1 protein purified from Expi293 cell production was tested with Western blot analysis using the same rabbit polyclonal serum.

### Animal immunizations

#### Pilot animal studies

Pilot animal DNA immunization studies were conducted in mice and non-human primates to compare the relative immunogenicity of different S-expressing DNA vaccine constructs (S-FL-wt, S-FL-opt and S-dTM-opt). Either 4–6 week old C57BL/6N mice or 1–2 year old rhesus monkeys were immunized three times (Weeks 0, 2 and 4) with 5μg DNA delivered each time by a Helio Gene Gun (Bio-Rad, USA). Serum samples were collected prior to the start of the study or 14 days after each immunization. Both mice and rhesus monkeys were housed in the Animal Research Center at the Institute of Medical Biology, Chinese Academy of Medical Sciences & Peking Union Medical College, in accordance with approved animal study protocol.

An additional pilot study was conducted in New Zealand White (NZW) rabbits. The rabbits were housed in the Animal Medicine facility at the University of Massachusetts Medical School in accordance with IACUC approved protocol. Rabbits were either immunized with DNA vaccines (S-FL-opt) three times (Weeks 0, 2 and 6) with 200μg DNA vaccine each time by the needle intramuscular injection (IM), or with two IM DNA immunizations (S-FL-opt or S-dTM-opt) at Weeks 0 and 2 followed by a one-time IM injection of 50 μg recombinant S1 protein vaccine at Week 6. Serum samples were collected prior to the start of the study or 14 days after the 3rd immunization.

#### Optimal vaccination design studies

The relative immunogenicity of different vaccination designs (S-FL-opt DNA vaccine, recombinant S1 protein, or co-delivery of S-FL-opt DNA vaccine and S1 protein) was further studied in the NZW rabbit model. All animals received three intramuscular (IM) needle immunizations at Week 0, 2 and 6 with fixed dosing: 200 μg S DNA vaccine and 50 μg S1 protein vaccine, delivered either alone or in combination (DNA and protein/adjuvant injected at the same time in separate sites). Serum samples were collected prior to the start of the study or 14 days after the 3rd immunization.

#### Non-human primate (NHP) immunogenicity and protection study

Groups of randomly assigned 1–2 year old rhesus monkeys (3–4 animals/group) were immunized three times at Weeks 0, 2 and 8 with one of the following vaccination regimens: S DNA vaccine pCW1093 alone (2 mg each time), recombinant S1 protein alone (100 μg each time), or co-delivery of S DNA vaccine pCW1093 and S1 protein at the same time but at separate sites, all delivered by intramuscular needle injections. The control animals received saline injections. Peripheral blood was collected prior to the start of the study and 7 days after each immunization for routine blood biochemical tests and SARS-CoV-2 specific immune responses tests.

A challenge study was conducted at 4 weeks after the third immunization by directly inoculating the rhesus monkeys with 5 × l0^6^ TCID50 of SARS-CoV-2 virus through the intratracheal route under anesthesia. At seven days after challenge, all animals were euthanized, the viral load in the different tissue was detected, and a pathological examination was conducted.

### Virus and cell line

SARS-CoV-2 strain BP16 was isolated from the sputum of a COVID-19 patient in Kunming, Yunnan, and amplified in Vero cells. The viral genome was extracted and subjected to nanopore sequencing (Nextomics Bioscience, Wuhan). The BP16 complete genome contains two mutations, C8782T and T28144C, in align with Wuhan-Hu-1. The former is a silent mutation, and the latter results in an amino acid difference in the ORF8 (L84S). BP16 was used in the neutralization and challenge assay. Vero cells were used for the production and titration of SARS-CoV-2 stocks. Vero cells were maintained in Dulbecco’s modified Eagle’s medium (DMEM, Corning), supplemented with 10% fetal bovine serum (FBS, Gibco), 100 IU/mL penicillin, and 100 μg/mL streptomycin, and incubated at 37°C, 5% CO_2_. The SARS-CoV-2 virus titer was determined by a micro-dose cytopathogenic effect (CPE) assay. Serial 10-fold dilutions of virus-containing samples were mixed with 2 × 10^4^ Vero cells and then plated in 96-well culture plates. After 5 days of culture in a 5% CO_2_ incubator at 37°C, cells were checked for the presence of a CPE under a microscope. Titers for SARS-CoV-2 were resolved by a 50% tissue-culture infectious doses (TCID50) assay.

### ELISA

The 96-well ELISA plates (Corning, USA) were coated with 0.2 μg/well S1 protein in 100 μL coating buffer(15 mM Na_2_CO_3_ and 35 mM NaHCO_3_, pH 9.6) and incubated at 4°C overnight. Plates were washed in PBST (0.5% TWEEN-20/PBS) and blocked using 2% BSA/PBST for 1hr at 30°C. Serially diluted serum samples were added and incubated for 1hr at 30°C. Plates were washed and horseradish peroxidase-conjugated goat anti-mouse IgG or anti-rabbit IgG (Invitrogen, USA) or horseradish peroxidase-conjugated goat anti-monkey IgG (Santa Cruz Biotechnology, USA) was added to all wells for 1hr at 30°C. The reaction was developed using TMB substrate (Makewonderbio, Beijing, China) and determined at 450 nm by a microplate reader. The S-specific IgG titers were determined by the end titration using a reciprocal of the last serum dilution that occurred when the OD value was still 2-fold greater than in the pre-bleed.

### Neutralization antibody assays

Two neutralization assays were used in the current report. The first one was conducted at IMB based on the neutralizing activities against real SARS-CoV-2 virus infection to Vero cells. In this assay, mouse or NHP serum samples collected from immunized animals were heat-inactivated at 56°C for 30 min and serially diluted with virus dilution medium at a starting dilution of 1:4 and then serially diluted 2-fold up to the required concentration. An equal volume of challenge virus solution containing 100 TCID_50_ virus was added, followed by 1 h of incubation at 37°C. Vero cells (2 × 10^4^ cells) were then added to the serum-virus mixture and the plates were incubated for 5 days at 37°C in a 5% CO_2_ incubator. Cytopathic effect (CPE) of each well was recorded under microscopes and the neutralizing titer was calculated by the dilution number of 50% protective condition. A neutralization antibody potency <1:4 is negative, while that >1:4 is positive.

The second neutralization assay is a pseudotyped virus based assay conducted at University of Massachusetts Medical School (UMMS). The pseudovirus system used is similar to those reported in literature [33]. The heat-inactivated immune rabbit serum samples were serially diluted at a starting dilution of 1:20 with 2-fold serial dilutions in 55 μl of volume. An equal volume of SARS-CoV-2 pseudovirus (100 TCID_50_/mL) was added, followed by a 1 h incubation period at 37°C. Then 100 μl of the serum/virus mixture was added to the 96 well plates pre-seeded with 1 × 10^4^ Vero-E6 cells per well. After the plates were incubated for 24 h at 37°C with 5% CO_2_, 100 μl/well fresh media was added. At 48 h after infection, cells were washed with PBS and then lysed using passive lysis buffer. The luciferase activities were developed with Luciferase substrate (Promega) and read. Neutralization was calculated as the percent change in luciferase activity in the presence of pre-immune sera versus that of luciferase activity in the presence of immune sera [(Pre-immune RLUs−Immune RLUs)/(Pre-immune RLUs)]×100. The NAb titers were determined at the serum dilution with 50% neutralization.

### ELISpot assay

Immunized macaque PBMCs were isolated to evaluate the antigen-specific T cell responses by ELISpot^PLUS^ (ALP) kits (Mabtech, Sweden). The ELISpot plates were incubated with 200 μl/well of serum-free media for 30 min at room temperature. Then 50 μl/well of pooled peptides (5 μg/peptide/mL) or S1 protein (20 μg/mL) in serum-free media and 50 μl/well of macaque PBMCs at 3 × 10^5^ cells/well were added. The plates were incubated for 16 h at 37°C with 5% CO_2_. After the plates were washed with pre-chilled water and PBS for 5 times, the plates were detected with conjugated anti-cytokine antibodies.

For macaque IFN-γ detections, biotinylated-anti-monkey IFN-γ at 1:1000 dilution in PBS with 0.5% FBS was added at 100 μl /well and incubated for 1 h at room temperature. Following wash, the plates were further incubated with 100 μl /well of ALP-conjugated-Streptavidin at 1:1000 dilution for 1 h at room temperature. Following washes with PBS for 5 times, the plates were developed with 100 μl /well of BCIP/NPT-plus substrate for 5 min in dark and washed with water and air-dried. For macaque IL-4 detection, the plates were directly incubated with 100 μl/well of ALP-conjugated-anti-human-IL-4 at 1:1000 dilution for 1 h at room temperature. Following washes with PBS for 5 times, the plates were developed with 100 μl /well of BCIP/NPT-plus substrate for 5 min in the dark and washed with water and air-dried. The immune spots in the ELISpot plates were counted using ELISpot reader (CTL, USA) and the final sport-forming units (SFUs) were calculated as spots/million cells.

### Realtime-RT-PCR assay

Tissues were homogenized in TRNzol universal reagent by TGrinder H24(TIANGEN, China) and RNA was extracted using Direct-Zol RNA Miniprep kit (ZYMO RESEARCH). Viral gRNA was reverse transcribed and amplified by One Step PrimerScript RT-PCR Kit (TakaRa) using Ligtcycler 480II Real-Time PCR System (Roche) according to manufacturer’s instructions. Viral loads were calculated as viral RNA copies per mL or per mg tissue and the assay sensitivity was 100 copies. The target for amplification was SARS-CoV2 N (nucleocapsid) gene. The primers and probes for the targets were: N-F:5’- GGGGAACTTCTCCTGCTAGAAT-3’; N-R: 5’- CAGACATTTTGCTCTCAAGCTG -3’; N-P: 5’-VIC-TTGCTGCTTGACAGATT-BHQ1-3’

For quantification of viral loads by RT-PCR, a standard curve of Ct values to the copy number of viral RNA is generated with serial 10-fold dilutions of RNA transcribed from recombinant plasmid pcDNA3.1-nCoV N in vitro with a known copy number. The viral loads of each sample were converted with Ct value and the standard curve. Statistical analysis was performed by LightCycler 480 Software.

### Histopathological analysis

The collected tissue sections (3 mm thickness) were fixed with 4% formaldehyde for 1 week. The fixed tissues were further dehydrated before being sliced into 2–3 μm thick sections and flattened on slides in warm water (40°C). The slides were further dried and dewaxed at 60°C, then stained with hematoxylin for 3–5 min, differentiated with hydrochloric acid aqueous solution followed with aqueous ammonia solution, and stained with eosin for 5 min after dehydration. The slides were then sealed with neutral gel.

### Statistical analyses

Analysis of virologic and immunologic data was performed using GraphPad Prism 8.4.2 (GraphPad Software). Comparison of data between groups was performed using two-sided Mann–Whitney tests. Correlations were assessed by two-sided Spearman rank-correlation tests. The student *t*-test was used to compare the antibody titers between groups. *P*-values of less than 0.05 were considered significant.

## Results

### Overall study design and S antigen selection

Our previous studies have demonstrated that a protein boost following DNA prime can lead to higher levels of antibody responses and the production of higher avidity antibodies than either DNA or protein immunization alone [34, 35]. This is presumably due to the fact that DNA immunization is good at inducing an antigen-specific B cell response while protein immunization can further stimulate activated antigen-specific B cells to produce large amounts of desired antibodies. This heterologous DNA prime-protein boost strategy has been highly immunogenic in human studies with HIV or influenza vaccines [36–39]. We have adopted the same concept in the current study to test whether co-delivery of DNA and protein COVID-19 vaccines can achieve the same level and quality of protective immune responses as the sequential DNA prime and protein boost approach. The co-delivery approach will be more practical for the large scale human applications.

**Figure 1.**
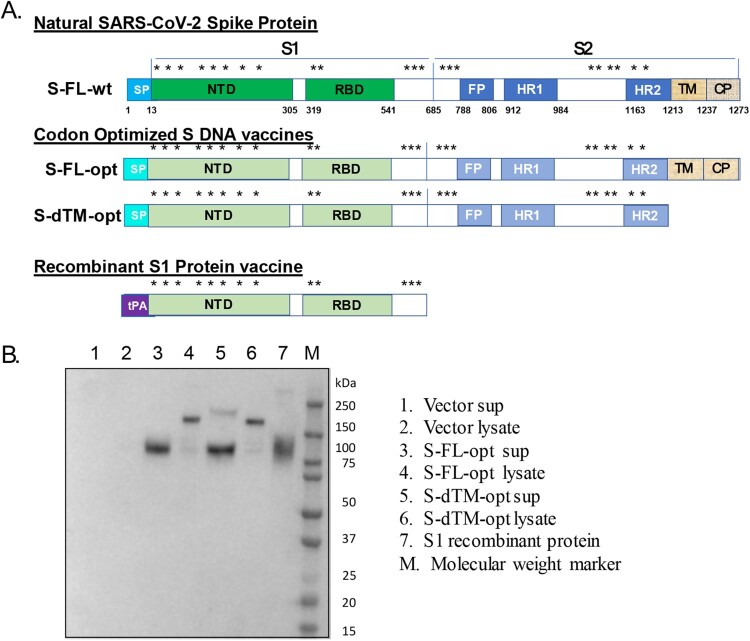
(A) Designs of SARS-CoV-2 spike DNA and protein vaccines. In addition to the wild type S gene insert (wt), two versions of codon optimized (opt) S DNA vaccines were produced: full length S insert (FL) and truncated S insert without transmembrane and intracellular components (dTM). For the expression of recombinant S1 protein, the signal peptide of tissue plasminogen activator (tPA) replaced the nature S protein signal peptide (SP). (B) Western blot analysis to examine the expression of S DNA vaccines and recombinant S1 protein vaccine. 293 T cells were transiently transfected with either S-FL-opt or S-dTM-opt DNA plasmids and either the culture supernatant (Sup) or cell lysate (lysate) was harvested 72 h later. Recombinant S1 protein was produced from Expi293 cells and purified by HisTrap HP. S1 specific rabbit polyclonal serum L295-IV was used as the detecting antibody.

Spike protein (S) of SARS-CoV-2 was selected as the antigen for this COVID-19 vaccine study based on our previous work on a DNA vaccine against the SARS virus more than 15 years ago [40] and recent literature regarding COVID-19 vaccine studies [6, 8–10]. However, different forms of S protein have been proposed in COVID-19 vaccine studies, including the full-length S antigen and various truncated forms of S antigens or stabilized pre-fusion state S antigens [10, 33, 41]. In our first animal experiment, immunogenicity of two versions of candidate S DNA vaccines was compared, not in stabilized pre-fusion state. S-FL-opt encodes the full-length S gene to express the exact same amino acid sequences as the natural S protein from the SARS-CoV-2 virus (Figure 1(A)). The only difference between the expressed sequences and the natural S protein is that wild type S gene nucleic acid sequences (-wt) are replaced with the codon optimized S gene sequences (-opt) using the approach we previously reported for SARS and influenza DNA vaccines [40, 42]. The other S DNA vaccine design is S-dTM-opt, which is similar to codon-optimized S-FL-opt but with the truncation of transmembrane and cytoplasmic domains of S protein (Figure 1(A)). The expression of S antigens by both DNA vaccines was confirmed using in vitro transfection of these DNA plasmids in 293 T cells followed by Western blot analysis (Figure 1(B)).

**Figure 2.**
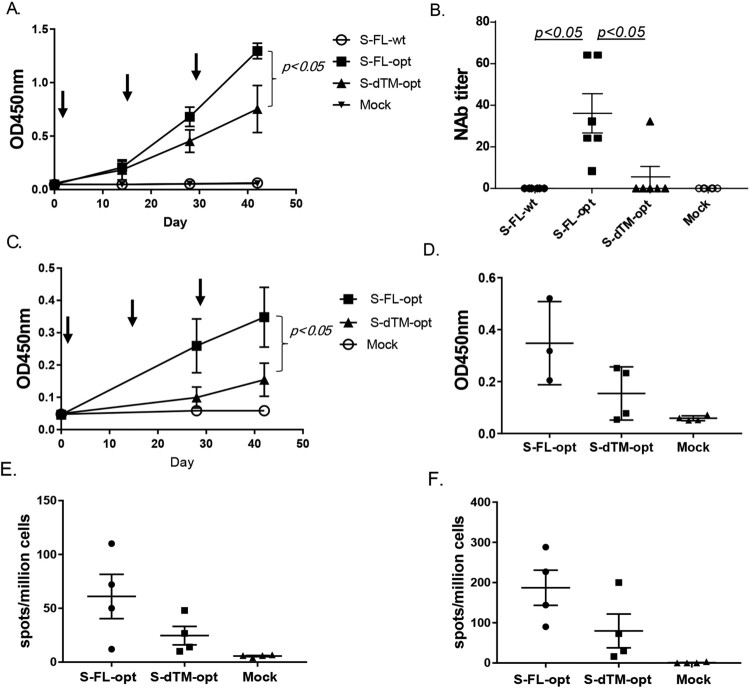
Pilot immunogenicity study of codon optimized and wild type S-expressing DNA vaccines. Individual mouse (A–B, *N* = 6 per group) or monkey (C–F, *N* = 4 per group) received three DNA immunizations as indicated by arrows using the gene gun delivery approach. The mock group received the empty DNA vaccine vector as the negative control. ELISA titers are shown as the average OD of each group (A, C) or end titration titers at the peak level Day 42 (D). Neutralizing antibody responses (NAb) (B) or IFN-γ (E) and for IL-4 (F) T cell responses are shown from each animal at the peak level on Day 42.

The relative immunogenicity of S-FL-opt and S-dTM-opt DNA vaccines was studied in multiple animal models. In C57BL/6N mice using gene gun delivery, both S-FL-opt and S-dTM-opt DNA vaccines elicited S-specific serum antibody responses and the titers increased following each immunization (Figure 2(A)). The peak level antibody responses after three immunizations were statistically different, with much higher titers in the S-FL-opt group than in the S-dTM-opt group. Meanwhile, mice received either the DNA vaccine encoding the wild type full length S gene sequences (S-FL-wt) or the saline injection (mock) did not have detectable S-specific antibody responses (Figure 2(A)). Consistent with the binding antibody data, immune sera from the S-FL-opt group had higher neutralizing antibody (NAb) titers than the S-dTM-opt group (*p* < 0.05) and no NAb was detected in either S-FL-wt or mock groups (Figure 2(B)). Overall, the NAb levels were low in the mouse model (∼1:20 to 1:60) when S-expressing DNA vaccines alone were tested.

In a pilot non-human primate (NHP) study using gene gun delivery, both the temporal development and the peak level serum S-specific IgG titers in S-FL-opt group were significantly higher than in S-dTM-opt group (*p* < 0.05) (Figure 2(C,D)). The NAb responses elicited by either of two S-expression DNA vaccines were low or barely detectable (data now shown), but the full-length S antigen design (S-FL-opt) was shown with ELIspot analysis to be able to induce higher levels of IFN-gamma and IL-4 responses than S-dTM-opt (Figure 2(E,F)).

### Immunogenicity of prime-boost vs. co-delivery of DNA and protein vaccines

With the identification of the optimal S-expressing DNA vaccine, a recombinant S1 protein was produced in parallel from transiently transfected Expi293 cells in order to be used for testing the DNA and protein combination vaccine strategy. The design of the S1 protein encoding gene is shown in Figure 1(A). In this design, a tissue plasminogen activator (tPA) leader replaced the natural signal peptide sequence of the S protein from SARS-CoV-2 with the goal of optimizing the production of a secreted S1 protein. This has been shown previously with other viral proteins [43]. The entire S1 protein sequence, including the receptor binding domain (RBD), is preserved in this design as in the original virus. It is now known that the production of full length SARS-CoV-2 S recombinant protein is technically challenging, as it is unstable and difficult to achieve a high yield of purified full-length recombinant S protein [44–46]. Because the RBD is considered the major target for protective antibody responses, we hypothesized that the S1 protein, instead of the full length S protein, should provide the same boosting effect to focus at the RBD region in a host primed with the full length S DNA vaccine. The recombinant S1 protein used in this pre-clinical study was partially purified by a research lab-based production process as shown in Lane 7 in Figure 1(B).

**Figure 3.**
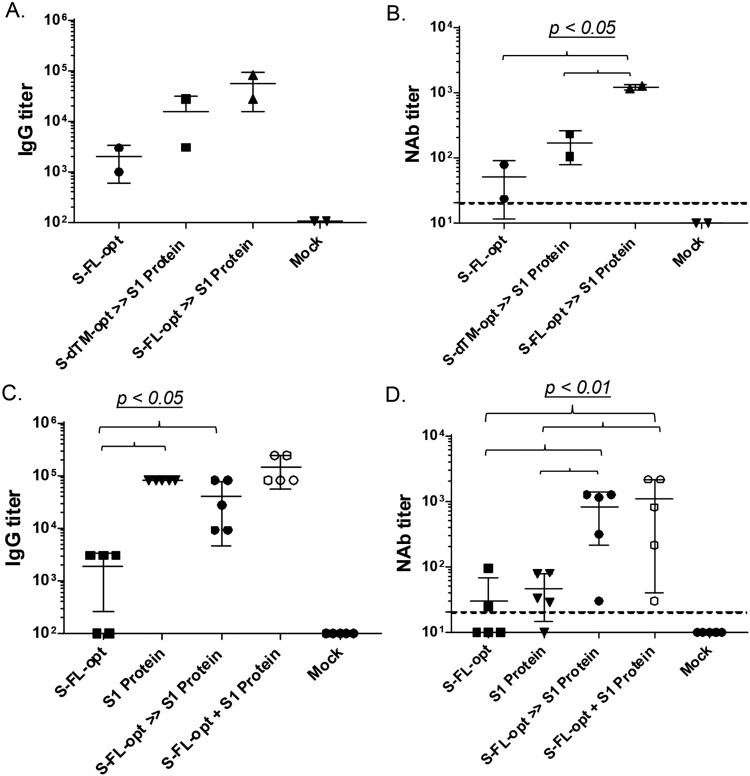
Relative immunogenicity studies in NZW rabbits. Animals were immunized three times at Weeks 0, 2 and 6 by intramuscular needle inoculations. Peak level (2 weeks after the last immunization) S-specific IgG titers (A & C) and NAb responses against pseudovirus (B & D) were measured either among DNA alone and DNA prime-protein boost approaches (A & B) or among DNA alone, protein alone, DNA prime-protein boost and co-delivery of DNA and protein approaches (C & D).

A study was conducted in New Zealand White (NZW) rabbits to test the immunogenicity of DNA and protein combination vaccine design. Both DNA and protein vaccines in this study were delivered by the traditional needle intramuscular injection (IM) because IM will be the route for human use of our DNA/protein vaccines. An adjuvant Alum was included in the protein formulation throughout the current study. Animals were immunized either with DNA vaccine alone (S-FL-opt), or with the DNA prime-protein boost (in this part of the study each animal was given the same S1 protein boost after priming with one of the two S DNA vaccines, either S-FL-opt or S-dTM-opt). The results clearly demonstrate that the protein boost is highly effective in eliciting much higher S-specific IgG responses than the DNA vaccine alone. The protein boost was able to further elevate the antibody titers in animals primed with the less optimal DNA vaccine S-dTM-opt more than in those receiving only the optimal DNA vaccine S-FL-opt. However, after the S1 protein boost, the titers in those primed with the optimal DNA vaccine S-FL-opt were still higher than those primed with the less optimal DNA vaccine S-dTM-opt (Figure 3(A)). The prime-boost groups showed easily detectable NAb responses and less variation between animals within the same group. The S-FL-opt prime followed by S1 protein boost had the highest titers of NAb (Figure 3(B)). These data indicate that priming with the optimal DNA vaccine design is critical, especially to the induction of high level NAb, and that the protein boost can further maximize the level of neutralizing antibody responses.

We next tested the relative differences in immunogenicity between the sequential and the co-delivery of full-length S-expressing DNA and S1 protein vaccines in the NZW rabbit model. The co-delivery immunization schedule is reported to be highly immunogenic in other report [47] and is easy to implement in large human populations because there is no need to track when a DNA or a protein vaccine component should be administered as in a sequential prime-boost design. Rabbits receiving the sequential DNA prime and protein boost vaccines (DNA>>Protein) had much higher S-specific IgG responses induced than the DNA alone group, but only slightly higher than the protein alone group (Figure 3(C)). However, serum NAb titers in the prime-boost group were much higher than protein alone groups (Figure 3(D)), supporting the value of DNA prime as we previously reported for HIV vaccine studies [34, 35]. It is possible that the DNA vaccine component is able to elicit highly conformational antibody responses than protein vaccines and such antibody responses are critical for the functional antibody activities such as the neutralizing antibodies [48, 49]. Furthermore, the co-delivery of DNA and protein vaccines (DNA + Protein) was equally immunogenic to the sequential DNA prime-protein boost approach in eliciting S-specific IgG antibody responses (Figure 3(C)). NAb responses were also very similar between sequential and co-delivery approaches (Figure 3(D)).

### Protection against SARS-CoV-2 challenge in a non-human primate model

**Figure 4.**
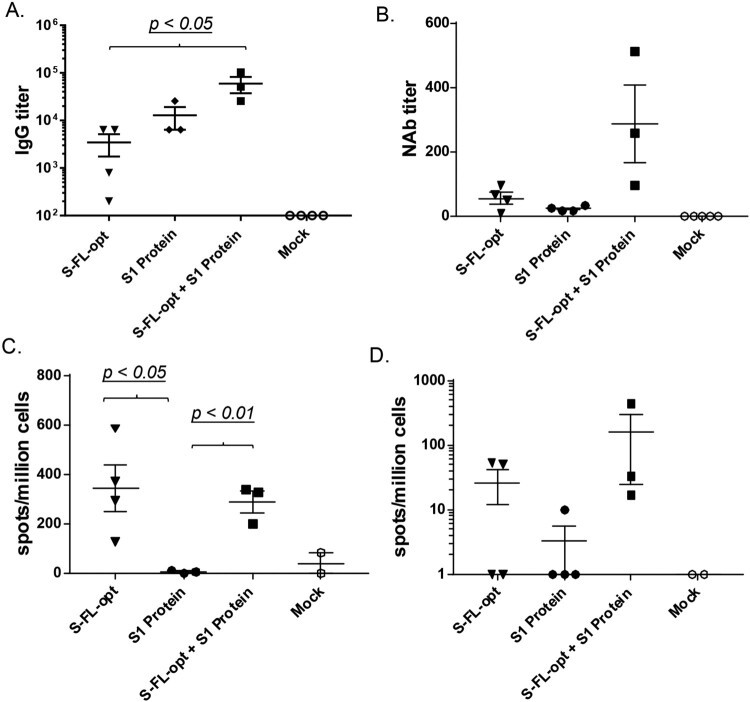
Non-human primate immunogenicity and protection study. Animals were immunized three times at Weeks 0, 2 and 8 by intramuscular needle inoculations. Peak level (2 weeks after the last immunization) S-specific IgG titers (A), NAb responses (B) against wild type SARS-CoV-2 virus by CPE assay and S-specific IFN-γ (C) and S-specific- IL-4 (D) responses were measured.

Based on the results from the above pilot animal studies, the co-delivery of DNA and protein vaccines approach (DNA + Protein) was selected as the chosen immunization design for our candidate COVID-19 vaccine and further tested against live SARS-CoV-2 viral challenge in a non-human primate (NHP) protection study. Similarly to the preliminary rabbit study, rhesus macaques received either DNA alone, protein alone or co-delivery of S-FL-opt DNA vaccine and recombinant S1 protein vaccine by IM injection at Weeks 0, 2 and 8. Co-delivery of DNA and protein vaccines showed higher peak level S-specific IgG responses than DNA alone or protein alone groups (*p* < 0.05 in both cases) (Figure 4(A)). The co-delivery group also elicited the highest NAb activities among three vaccine groups (Figure 4(B)). Regarding T cell immune responses, both the DNA alone and co-delivery groups were able to elicit robust IFN-gamma and IL-4 responses at much higher levels than those detected in group vaccinated with the protein alone (Figure 4(C,D)). Our data validated the long-standing concept that DNA vaccines are effective in eliciting T cell immunity [50–52] while protein alone vaccines have poor T cell immunogenicity.

**Figure 5.**
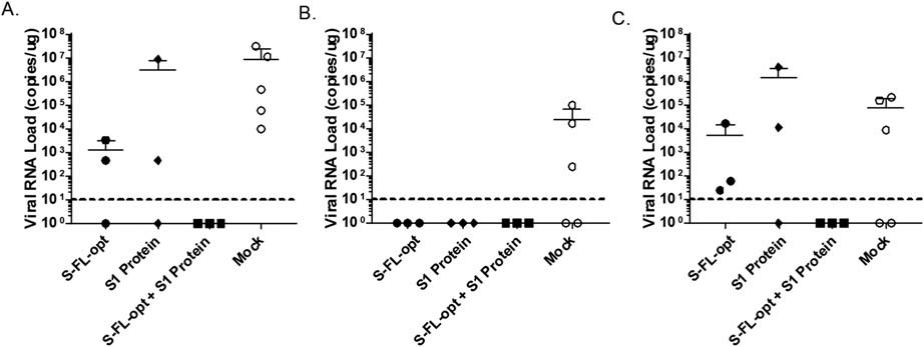
Viral RNA load detected at various NHP tissues after challenge. Monkeys immunized with various vaccine approaches as described in Figure 4 were challenged with live SARS-CoV-2 virus through intratracheal route and animals were sacrificed 7 days later and viral load (copies /μg) was measured in lung (A), lung lymphoid tissue (B) and trachea (C).

Animals in this NHP study were further challenged with the live SARS-CoV-2 virus through the intratracheal route. For animals in the mock group, high levels of the virus were detected in trachea, lung and lung lymphoid tissue. In animals receiving the DNA vaccine alone or protein vaccine alone there was no virus detected in lung lymphoid tissue, but there were positive virus detections in both the trachea and lungs, although the viral loads at these locations were slightly lower in some animals in these two groups than those in the mock group (Figure 5(A–C)). In contrast, the animal group receiving the co-delivery of S-FL-opt DNA and recombinant S1 protein vaccines achieved full protection. No virus was detected in trachea, lung or lung lymphoid tissues (Figure 5(A–C)).

**Figure 6.**
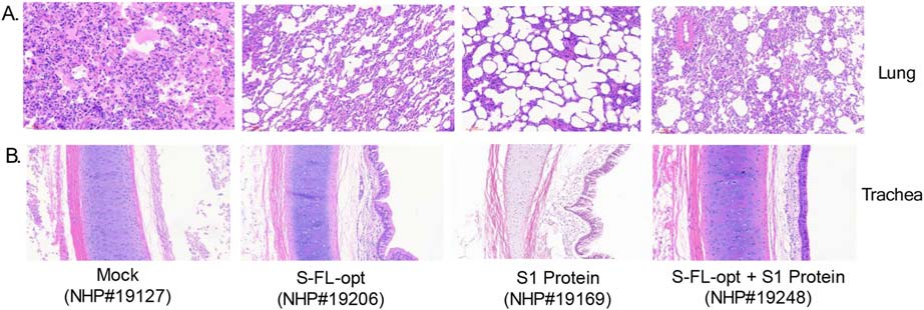
Histology analysis of key organ tissue samples including lung (A) and trachea (B) from non-human primates receiving different vaccination regimens in the study (individual rhesus macaques numbers used in the study are shown).

Histology analysis of sacrificed animal lung samples showed severely disrupted pulmonary alveoli structure along with massive infiltration of inflammatory cells and large amount of exudation in the mock group (#19127). For S1 protein alone group animal (#19169), mild alveola structure disruption was observed with changes consistent with emphysema. In contrast, only increased cell infiltrations in interstitial tissue especially lymphocytes were observed in either S-FL-opt (#19206) or S-FL-opt + S1 protein (#19248) immunized animals (Figure 6(A)). Similarly, the trachea’s mucosal surface was severely damaged with severe edema in the mock group (#19127), cell falling and mild edema in S1 protein group (#19169), and some cell falling in S-FL-opt group (#19206). In contrast, no observable changes in trachea tissue in DNA + protein group (#19248) (Figure 6(B)). In summary, our data demonstrated the full protection of the novel co-delivery of DNA and protein COVID-19 vaccines against SARS-CoV-2 challenge in a non-human primate model based on the strong immunogenicity of antibody and T cell responses.

## Discussion

A safe and efficacious SARS-CoV-2 vaccine is needed to end the global COVID-19 pandemic. Two mRNA based COVID-19 vaccines have already received emergency use authorization (EUA) in the United States and European Union for large human population use, and still other COVID-19 vaccine candidates are in Phase III human efficacy trials with great potential for regulatory approval in the near future. Given the urgent need for these vaccines, a number of important questions have not yet been answered. These questions include that of their potential for long term protection and safety profiles in large human populations. Both will need close follow-up over time. At the same time, additional vaccine platforms have continued to progress, and information from these studies will offer valuable knowledge in addition to those provided by early generation COVID-19 vaccines.

In this report, we demonstrate the high immunogenicity of combined DNA and protein COVID-19 vaccines when delivered at the same time. While it is demonstrated in literature that the vaccination approach of DNA prime and protein boost is a powerful immunization strategy, this report further confirms that co-delivery is as equally immunogenic as sequential immunization. This conclusion is based on both binding and functional antibody responses. Furthermore, this study is the first time that the co-delivery of DNA and protein vaccines was able to elicit full protection against an acute emerging viral infection like SARS-CoV-2 in an NHP model while DNA alone or protein alone vaccine components were demonstrated to be less effective. Since most of the NHP studies with other leading COVID-19 vaccines only demonstrated partial viral reduction and not full protection [6, 18, 27–29, 33, 41], the DNA and protein co-delivery vaccine strategy may have the potential to generate stronger and longer-lasting immune protection than other leading vaccines even though NHP study results may not be directly translated into human efficacy.

As we previously reported using HIV-1 or influenza vaccine models, DNA immunization can use both innate and acquired immunity mechanisms to induce the development of antigen-specific B cells, especially the germinal center B cells which are the basis for high affinity antibody responses [26, 53]. It is now known that SARS-CoV-2 infection does not establish long-lasting antibody responses in patients who had mild clinical symptoms, which implies that a successful COVID-19 vaccine needs to elicit a stronger response than natural infection, and a long-lasting immune response that includes potent S-specific memory B cell responses. Our approach will greatly facilitate this process by including the DNA component. The inclusion of a DNA vaccine component can serve two important purposes: first, to improve the quality of antibody responses such as the levels of NAb, due to the ability of DNA vaccines to induce better antibody responses against conformational epitopes [48, 49], and second, to elicit high levels of antigen specific memory B cells through stronger activation of germinal center B cell development than that which occurs with protein based vaccines [26, 54].

In the history of nucleic acid vaccines, DNA vaccines were the first to enter human studies. It was soon realized that the immunogenicity of DNA vaccines in humans was low when used alone as naked plasmids [55, 56]. A wide range of strategies have been tested to improve the immunogenicity of DNA vaccines, including lipid formulation, the use of adjuvant plasmids encoding for cytokines, and gene gun or electroporation delivery. These were helpful but also added complexity, cost, or safety concerns. On the other hand, the prime boost strategy of combining a DNA vaccine with another vaccine modality such as a recombinant protein vaccine [31, 34, 35], inactivated vaccine [37, 57], or even live attenuated vaccines [58] achieves the most desirable immune responses as such combinations are able to maximize the benefits of two types of vaccines while overcoming their potential drawbacks. The technologies for other vaccines already exist and are well accepted, making it straightforward to combine these existing vaccines with DNA vaccines. The sequential prime-boost can generate logistical issues because the vaccine recipients and their caregivers need to document and track when to give the prime component and when to give the boost component, and the co-delivery approach will make the vaccination process simpler and easier to manage.

Immunologically, the combination of DNA and protein vaccines enables to generate high avidity antibody responses as we previously reported [34] which suggested an efficient antibody maturation process that is important for a high affinity and long lasting protective antibody response. Given the reports of antibody responses after SARS-CoV-2 infection being low level and quickly decreasing, a successful COVID-19 vaccine would need to induce more persistent immune responses. At this point, it is difficult to envision the complete eradication of SARS-CoV-2 in the global human population. Therefore, a long lasting immune response is critical to achieve a persistent protection via a vaccine. Either DNA or protein alone may not achieve such immunological effects.

Another novelty of our study is that there is some, but not entire, overlap between the antigens used in the DNA and protein vaccine components. As shown in both this report and other COVID-19 vaccine studies, the use of the full-length S antigen is important in inducing the most robust protective immune responses. It was found that the full length S antigen induces better protective immune responses than the receptor binding domain (RBD) antigen [33, 59]. Different from the two EUA approved mRNA vaccines expressing modified prefusion-stabilized version of S protein (S-2P), the full-length DNA vaccine used in this study expresses the wild type form of S protein in order to preserve the native conformation. The relative immunogenicity of DNA vaccines expressing prefusion-stabilized versions of the S protein in comparison to those expressing the native form warrants further evaluation. While it is easy to produce nucleic acid vaccines with the full length S, it is quite challenging technically to produce the full length recombinant S protein. In our study we used the S1 recombinant protein, which alone would not induce high level NAb but was able to boost the high level NAb on the basis of DNA prime or co-delivery. Although the exact mechanism of this mismatched boost needs to be investigated, this opens the door for the design of vaccines against highly conformational antigens.

Our results provide another successful example of the heterologous immunization strategy in which different vaccine modalities are combined to deliver the same antigens [36]. The scientific significance of this extends beyond the use of DNA vaccines. For example, inactivated vaccines may have relatively low immunogenicity and viral vector vaccines can only be used once or twice to avoid anti-vector immunity. Yet this study demonstrates that the combination of inactivated COVID-19 vaccines with viral vector based COVID-19 vaccines can be expected to enhance both immunogenicity and protection. The COVID-19 vaccine developed by Russian scientists using two adenovirus vectors in a sequential delivery format is another example of such a heterologous prime boost and was shown to improve the immunogenicity of both vaccines by reducing the anti-vector immunity [60]. Much work is still needed in order to fully take control of COVID-19 pandemic, and our study offers another option for improving the current global situation by developing safer and more effective COVID-19 vaccines with long-lasting immunity.
